# Extradural Clinoidectomy in Clinoidal Meningiomas: Analysis of the Surgical Technique and Evaluation of the Clinical Outcome

**DOI:** 10.3390/tomography8050197

**Published:** 2022-09-23

**Authors:** Luigi Sampirisi, Luca D’Angelo, Mauro Palmieri, Alessandro Pesce, Antonio Santoro

**Affiliations:** 1 Neurosurgery Division, Santa Maria Goretti University Hospital, Via Lucia Scaravelli, 04100 Latina, Italy; 2 Human Neurosciences Department, Neurosurgery Division, “Sapienza” University, AOU Policlinico Umberto I, 00185 Rome, Italy

**Keywords:** clinoidal, clinoidectomy, meningiomas, visual function

## Abstract

The surgical treatment of clinoidal meningiomas is currently still discussed in the literature. Different surgical approaches have been proposed and evaluated, in multiple studies, in order to improve the surgical outcomes. The aim of this study is to evaluate the advantages of extradural clinoidectomy in the context of tumor removal radicality for visual function improvement. A retrospective analysis was performed on 74 patients—of which 26 patients with clinoidal meningiomas were in group III, according to Al Mefty classification—who underwent surgery at the Policlinico Umberto I Hospital between 2000 and 2019. Further, extradural clinoidectomy was performed on 15 patients (Group A), and 11 patients underwent the pterional approach only (Group B). Additionally, visual impairment was present in all 26 patients before surgery. Next, visual function assessment was performed on all patients, both in presurgery and postsurgery. Radiological follow up was performed at 3 and 6 months, and then every 12 months. Gross Total Resection (GTR) was achieved in 13/15 (86.7%) patients who underwent clinoidectomy, and in 4/11 (36.4%) patients who did not undergo clinoidectomy. Visual function improvement was achieved in 12/15 (80%) patients who underwent clinoidectomy and in 4 of 11 (36.4%) who did not undergo clinoidectomy. According to our study, extradural clinoidectomy is the most suitable method for facilitating the gross total resection of clinoidal meningiomas. Our experience and data suggest that a higher rate of total resection and, subsequently, the best visual outcomes are achieved. Extradural drilling via the anterior clinoid process reveals a wider surgical corridor for meticulous tumor resection.

## 1. Introduction

Clinoidal meningiomas (CMs) derive from the meningeal membrane of the anterior clinoid process (ACP). The incidence rate ranges from 34% to 43.3% in all tumors involving the sphenoid wing [1]. Vision loss is the most common symptom and, depending on its anatomy and development, it can be preceded, or followed, by headache and nausea. When the optic chiasm or the optic nerve are compromised, visual field deficit, bitemporal hemianopsia, and scotoma may be present. Mass effect may cause exophthalmos or paralysis of the cranial nerves (III, IV, and VI CNs), especially when the superior orbital fissure and the cavernous sinus are involved. From a radiological perspective, hyperostosis of the ACP is a useful radiological sign in a brain CT scan. Further, fat suppression MRI shows the tumor extension in the orbit. Al-Mefty based his classification on microsurgical anatomy features, and it is now widely accepted. The classification is based on surgical microanatomy findings, tumor origin, and the adherence to the internal carotid artery (ICA). The classification differentiates three subgroups of such tumors [2,3,4,5]: Group I: Meningiomas which grow over the lower surface of the ACP and surround the ICA, just before it enters the arachnoid cistern. These meningiomas directly adhere to the arterial adventitia, without interfacing of the arachnoid sheets. In this anatomical condition, dissection from the artery is generally unfeasible. Group II: CM originating from the superolateral aspect of the ACP. When these tumors increase in size, they remain covered by the arachnoid sheets around the carotid cistern. Consequently, the tumor is separated from the ICA’s wall by the arachnoid, which prevents adherence to the adventitia. In this case, resection is easier. Group III: CM originating from the region of the optic foramen and extending into the optic canal become symptomatic early and are diagnosed before they reach large sizes, unlike type I and II. Considering tumor size, other authors have proposed a further division of these groups according to tumor mass (≤2 cm, between 2 and 4 cm and ≥4 cm).

The gold standard treatment is surgery. This is because radiotherapy can be harmful to visual apparatus, especially in small-sized lesions, and should be reserved for the management of biologically aggressive lesions with a faster growth rate. The goal of surgery should always be to conduct the most radical possible resection at first attempt [2,3,4,5] as recurring surgery can be arduous and associated with a higher risk of morbidity and mortality. The reported resection rate ranges from 43% to 91% [6,7], depending on tumor size, histology, and encasement pattern (Al Mefty group I or III). Extradural clinoidectomy is performed in a selected subgroup of patients, whose lesions have already invaded the optic canal, and requires proper optic canal unroofing and decompression. Via this unroofing, the EC could both improve the visual function outcome, decompressing the optic nerve and increasing the EOR, as well as facilitate access to a normally hidden residual disease.

The purpose of the present study is to evaluate the potential role of extradural clinoidectomy (EC) in increasing the extent of resection (EOR) and improving visual outcomes in a consecutive series of microsurgically treated patients suffering from Group III CMs. Since visual impairment is particularly common in this subgroup of meningiomas, special attention is paid to the visual function outcomes and the incidence of postoperative complications in order to define the safety effectiveness profile of this maneuver.

## 2. Materials and Methods

We retrospectively analyzed the clinical and radiological records of a cohort of 74 patients (51 females and 23 males) who received a radiological diagnosis of CM in our neurosurgery department between 2000 and 2019. All of these 74 clinoidal meningiomas were categorized according to the Al-Mefty classification. Further, only 26 were classified as type III and thus met the inclusion criteria of our investigation. These 26 patients were divided into two groups. Extradural clinoidectomy (EC) was performed in 15 patients (group A), and was not performed in the remaining 11 patients (group B). The decision of whether to complete the exposure with an EC depended on the involvement of the inferior and medial aspect of the ACP with or without the extension of the lesion around the optic nerve.

### 2.1. Radiological and Clinical Protocol

For each group, a postoperative MRI was performed at 3 and 6 months, and then every 12 months to check residual instances or recurrence of meningiomas. The postoperative brain MRIs were compared against the preoperative brain MRIs. Both MRIs were performed with a standard high-field magnet with the following sequences: T2w, FLAIR, isotropic volumetric T1-weighted magnetization-prepared rapid acquisition gradient echo (MPRAGE), and before and after intravenous administration of paramagnetic contrast agent. The greater diameters and the volume of the lesions were calculated and recorded. Furthermore, all patients underwent DVFT and VF examinations at the ophthalmologic clinic in Policlinico Umberto I University Hospital of Rome after 1, 3, and 6 months, and then every 12 months postsurgery. The visual field deficit was recorded and compared with the postoperative conditions.

### 2.2. Surgical Protocol and Technique: Extradural Clinoidectomy

A standard total intravenous anesthesia protocol with Propofol and Remifentanil was used. At the beginning of the procedure, before the skull clamp was positioned, local anesthesia was carefully applied to a regional total nerve block (including the supraorbital, auriculotemporal, and occipital nerves) of the scalp, the surgical wound course, and the intended Mayfield clamp pin sites. This was performed with the infiltration of ropivacaine with adrenaline.

Every patient was placed in the supine position, with a soft pad positioned under their ipsilateral shoulders, and their heads were rotated contralaterally. In both groups, a frontotemporal approach was adopted.

The ACP is an anatomical structure of deep surgical interest that presents an anatomical relationship with relevant neurovascular structures: internal carotid arteries (ICA); optic nerve ON (CN II the second cranial nerve); oculomotor nerves (CN III the third cranial nerve); superior orbital fissures (SOF); and cavernous sinuses (CS) [6,7]. After a pterional or orbitozygomatic craniotomy, the dura over the medial sphenoid wing is elevated from the medial sphenoid wing toward the ACP, which is covered by two layers of dura mater: the superficial periosteal dural layer and the inner meningeal layer. At the level of SOF, the inner layer contains the cranial nerves and the superficial periosteal dural layer forms a fibrous, thick fold of dura called the meningo-orbital band. This band is coagulated and cut. Additionally, this maneuver allows one to identify the whole ACP, the vascular structures, and to coagulate and cut the meningo-orbital artery to reduce tumor blood supply. Next, as SOF laterally and ON medially are important anatomic landmarks, the bone between these structures and the roof of the optic canal are subsequently drilled along their entire length. We use an ultrasonic aspirator (CUSA) with a specific bone tip to minimize the risk of thermal damage (see video). Finally, the optic strut is drilled and a microdissector is used to remove the apex of the clinoidal segment. As EC is completed, the paraclinoid segment of the ICA—which defines the anterior loop and branches of the ophthalmic artery—appears. In this region, the ICA leaves the roof of the CS through the proximal ring and enters the intradural compartment through the distal carotid ring, thus becoming the supraclinoid ICA. This maneuver allows for early proximal arterial control and a wider intradural corridor. Occasionally, the ACP is included with the sphenoid sinus and connected with the nasal cavity, thus producing the risk of a CSF leak. It is mandatory to repair this with muscle, fat, or sealant material. Once EC is performed, the attachment of the lesion is exposed and the tumor is subsequently removed intradurally with a standard microsurgical technique, thus availing one with an increased surgical corridor.

The execution of EC, when needed, allows one to obtain a wider surgical corridor and a better view of the neurovascular structures located in this area. Moreover, minor brain retraction is needed and it also possible to achieve extradural devascularization of the lesion. However, EC is a maneuver that can lead to possible complications due to its complexity: For instance, the high-speed drill could damage the surrounding structures, especially the II and III ipsilateral cranial nerves. Moreover, a higher risk of CSF leakage is connected to EC being executed. EC is a delicate surgical maneuver that requires knowledge, experience, and skill to perform safely; therefore, the learning curve related to its execution must be considered during preoperative planning.

For more information, see the video in the Supplementary Materials of an extradural clinoidectomy being performed for a case that is included in the present study.

### 2.3. Endpoint Variables

We recorded the incidence of postoperative complications (as a dichotomous variable), the visual function outcomes (as a three-step ordinal variable, namely 0: worsened; 1: unchanged; 2: improved), and the extent of resection (EOR). The EOR was evaluated by comparing the pre and postoperative T1w with gadolinium-enhanced sequences to check possible residuals. Since it is generally impossible to remove the basal dura surrounding the ACP (whenever the Simpson Grade of Resection reached at least the level of 1, 2, or 3), the resection was classified as gross total (GTR). Conversely, in the case of Simpson Grade of Resection 4, the resection was classified as gross total (STR). No biopsies (grade 5) were included in the present series.

### 2.4. Statistical Tests

The sample was analyzed with SPSS version 18. Further, comparisons between nominal variables were made with Fisher’s exact tests. The EOR was compared with one-way ANOVA analysis, contrast analysis, and post hoc tests. Continuous-variable correlations were investigated with Pearson’s bivariate correlation. The threshold of statistical significance was considered to be *p* < 0.05.

The data reported in the study have been completely anonymized. Due to the study’s retrospective nature, no randomization of treatment was performed. Further, there were no deviations in respect to the world-recognized gold standard treatments in the preoperative or postoperative treatments or diagnostic tools. The Institutional Review Board of our institution approved the informed consent of the patients. All the patients and/or their representatives issued written consent for the utilization of appropriate patient information for this study. Additionally, this study is consistent with the Helsinki Declaration for ethical principles in respect of medical research on humans.

## 3. Results

As previously stated, a total of 26 patients were classified as Type III CM and thus met the inclusion criteria: a total of 17 females and 9 males whose average age is 60.3 ± 12.5 (range 25–80) were included in the study. The average volume of the lesions was 11.84 ± 10.68 cm^3^. All relevant information concerning the cohort is summarized in Table 1.

Digital visual field tests (DVFT) were performed: 21 patients suffered from visual field (VF) defects as well as decreased visual acuity, whereas 5 patients only possessed a visual field defect. In particular, the in-depth examination of the VF revealed that 14 patients were affected by a concentric reduction in the VF, 7 patients by bitemporal hemianopsia, and 5 patients by retinal scotoma.

No differences regarding blood loss and postoperative length of stay were registered between patients who were treated with the execution of EC and those who were treated through a standard approach.

### Extent of Resection Analysis

**Figure 1:**
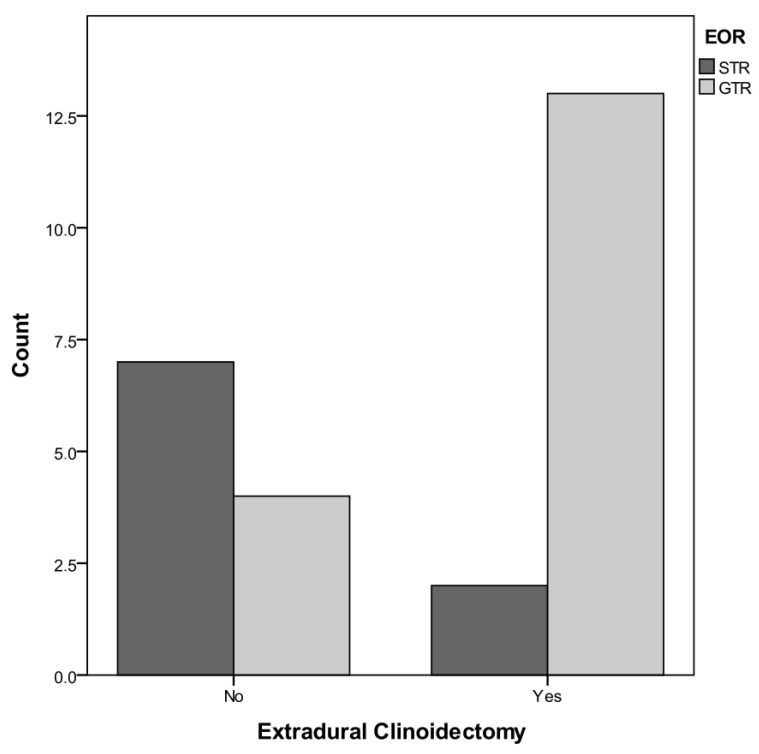
Confrontation between Groups A and B regarding the impact of extradural clinoidectomy on EOR.

Regarding the first objective of the present study—namely the EOR analysis in EC patients, as previously stated—Simpson I in CMs are not always feasible. Rather, it is impossible to completely remove the basal dura surrounding the ACP, and therefore a safe removal with, or even without, coagulation of the dural attachment (Simpson 2 or 3, respectively) may also be considered as a GTR, because the bulk of the tumor is resected and no macroscopic residual is left in situ. In the case of macroscopic tumor residuals, in respect to the anatomical relationship with the arteries, optic nerve, and/or the cavernous sinus, tumor removal was considered to be partial (Simpson 4). Our results highlight that: In group A (where EC was performed), we obtained a GTR in 13 patients (Figure 1 and a STR in 2 patients (Simpson 4); conversely, a GRT was reached in 4 patients from group B (where EC was not performed), with a partial asportation in 7 patients. To summarize, of those patients who underwent extradural clinoidectomy, a total resection of the tumor was achieved in 86.7%, whereas of those who did not undergo EC, a total resection of the tumor was achieved in 36.4%. As such, this data reaches the threshold for statistical significance (F = 0.0135, Figure 1).

**Figure 2:**
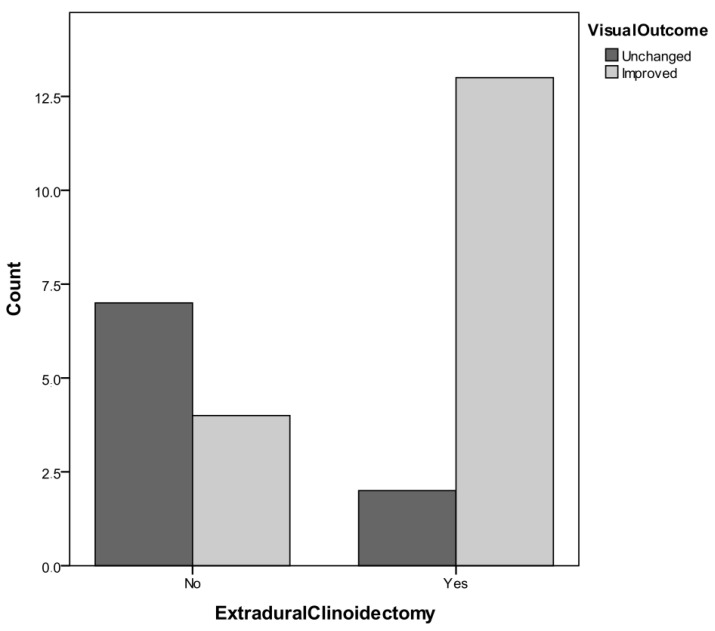
Confrontation between Groups A and B regarding the impact of extradural clinoidectomy on visual outcome.

Regarding the second aim of the study, our results outline that: In Group A (where EC was performed), 12 patients experienced an improvement of visual function and 3 patients were unchanged, whereas in group B (where EC was not performed), 4 patients experienced improved visual function postoperatively, and 7 patients were unchanged. Practically, 80% of the patients who underwent EC experienced a better visual outcome, whereas 36.4% of the patients who did not undergo EC achieved an improved visual outcome. Moreover, this result also reached the threshold for statistical significance (F = 0.0426, Figure 2).

In a total of four patients, there was an infiltration of the cavernous sinus. In such cases, the cavernous sinus was never surgically accessed to minimize the postoperative surgical morbidity profile. We recorded a total of three postoperative complications. Two patients experienced postoperative seizures and one patient experienced a postoperative CSF leak. There was no statistically significant difference in the incidence of complications between the two subgroups. Further, there were no postoperative deaths in our cohort either.

## 4. Discussion

Gross total resection is the surgical target for virtually all CNS meningiomas. In CM surgery, the achievement of a total resection is intimately associated with the visual outcomes, the risk to cranial nerves, and also vascular injuries. This is because CMs have a close relationship with vital neurovascular structures such as the internal carotid artery (ICA) and its branches, the optic nerve and chiasm, and the cavernous sinus. Due to the anatomical complexity of this region, surgery of CMs still represents a challenge for neurosurgeons, despite the advances in neurosurgery that have been achieved during the last few decades and which have significantly helped in decreasing the morbidity and mortality profile of such a complex surgery.

In the first instance, the choice of the proper surgical approach is essential it needs to be tailored according to the tumor size, the extension of neurological conditions, preexisting neurological conditions, and the specific anatomical features of the lesion and of the patient. A pterional approach has been suggested to deal with small meningiomas that have superolateral extension and cause mild neurological symptoms [5,8,9], while a fronto-orbitozygomatic approach is suggested instead for large meningiomas that have diffuse invasion of neurovascular structures [10]. The surgical technique of EC, however, has been advocated to minimize the residual disease in extensive and complex CM resections.

This technique, initially introduced by Dolenc and reserved for lesions involving the cavernous sinus, demonstrates major advantages and few complications. In our series, the use of EC in surgery for Group III CM produces a wider surgical exposure, thus providing significant advantages in terms of a higher GTR rate (86.7%) and also increased chances of experiencing a visual improvement (80%).

Removal of the ACP allows wider access, a better view of the surgical corridors, and clearer exposure of the neurovascular structures while also decreasing unnecessary brain tissue retraction. Further, the ICA and the optic nerve may be identified and controlled earlier in the procedure; consequently, tumor devascularization and optic nerve decompression is achieved. As this is achieved earlier, it is more effective and therefore decreases instances of optic manipulation and the risk of vascular injuries. Furthermore, when the tumor presents an intraorbital extension, the opening of the optic sheath is recommended to detach and mobilize the optic nerve from the tumor and to reduce the risk of intraoperative injury of the nerve.

In agreement with our results, the authors Lee, Marinello, and Giammattei [10,11,12] reported the critical role of EC in CM surgery in terms of increasing GTR while also lowering the morbidity rate. Visual impairment is frequently present in CM, especially in group III. The process of optic nerve damage includes ischemic insult, which is caused by a decreased blood supply and/or mechanical damage caused by direct compression [13]. Visual acuity deterioration, diplopia, ipsilateral hemianopsia are the most common complaints. Diplopia may be irreversible or show a gradual recovery depending on the combination of ischemic and mechanical injury on III, IV, and VI CNs, which are characterized by thinner fibers and a more delicate course.

Analyzing the literature, a postoperative visual function improvement is expected in 62% to 77% of patients [14,15,16,17], while in 33% to 47% of patients the visual function remains unchanged. Worsening of visual function occurs only in 1% to 5%. A better outcome is described by authors that performed an EC.

Some authors advise that an extradural approach [4,8,11,12,13] and unroofing of the optic canal [11] are useful techniques to minimize the risk of optic nerve injury. Our data outline that EC provides a visual outcome improvement in 80% of patients. In our experience, Group III CMs (where there is compressing of the optic nerve) become symptomatic in their earlier growth, and often the visual impairment is the only apparent symptom. An early resection may guarantee a total resolution of the visual complaint because the injury is still in a reversible phase.

Infiltration of the adjacent neurovascular structures and extension into the cavernous sinus are among the most important causes of partial resection and, subsequently, of increased risk of recurrence. In the recent meta-analysis of Gianmattei et al., a higher rate of recurrence is reported for skull-based meningiomas, especially in long follow up programs (>48 months) [12,14,15,16,17]. Such data could be improved with the selection of less aggressive surgical resection strategies in order to avoid risks of serious complications. Even though the GTR is the aim of CM, and in general of meningioma surgery, preservation of the surrounding nerves and blood vessels should be always kept as the absolute priority. Our clinical data demonstrate that patients who underwent an EC could obtain a higher resection rate and could thus experience a lesser rate of early and late recurrences.

However, a multidisciplinary approach in therapeutic strategy for CM has improved the prognosis. Radiotherapy is a safe and feasible treatment in residual or recurring tumors. In the past two decades, several series reported the important role of radiation therapy in the multimodal treatment of intracranial meningiomas [18]. Postoperative gamma knife radiosurgery and hypofractionated stereotactic radiotherapy on residual portions of a tumor or during a recurrence should offer a higher tumor control rate and a longer progression-free survival. Furthermore, a recent study reported that gamma knife radiosurgery as a primary treatment in small size (<3 cm) CM induced a regression of tumor volume and an improvement of visual deficits, although such findings are still limited experiences with respect to the traditionally confirmed role of surgery [19]. Radiotherapy is indeed not devoid of the risk of complications including optic nerve neuropathy, cranial nerve dysfunction, cognitive deficits, and seizures. Second-look surgery should be considered on a case-by-case basis since it has been previously reported to be associated with higher mortality and morbidity rates due to adherences and fibrous tissue, the more significant probability of CSF leaks, and/or intracranial bleeding [19]. The safer therapeutic approach should always be tailored to the precise features individual patients: any recurrence has a precise anatomical extension and, case by case, a second surgery or radiation treatment could be the better option.

## 5. Conclusions

Extradural clinoidectomy offers several advantages in clinoid meningioma surgery. It allows for the safe removal of tumors through early decompression, mobilization of the optic nerve, and earlier proximal control of the internal carotid artery. Moreover, it expands the surgical view, therefore increasing the chances of reaching total tumor resection while also decreasing brain retraction. Our retrospective analysis of group III CMs confirms the role of EC in improving both the visual function outcomes and in increasing the GTR.

## Figures and Tables

**Table 1:** Patient demographics.

Group	Extradural Clinoidectomy		Standard Approach		Total		*p* Value
Sex	M: 5	F: 10	M: 4	F: 7	M:9	F: 17	0.879
Age	58.8 ± 14.95		62.2 ± 8.3		60.3 ± 12.4		0.495
Tumor size	10.5 ± 9.2 cm^3^		13.6 ± 12.7 cm^3^		11.8 ± 10.7 cm^3^.		0.474
Preoperative visual deficit	Retinal scotoma: 0 Reduction visual field: 9 Bitemporal hemianopsia: 7		Retinal scotoma: 5 Reduction visual field: 5 Bitemporal hemianopsia: 1		Retinal scotoma: 5 Reduction visual field: 14 Bitemporal hemianopsia: 8		0.243
Extent of resection	Total: 13 Subtotal: 2		Total: 4 Subtotal: 7		Total: 17 Subtotal: 9		0.006
Complications	CSF leakage: 1 Seizure: 1 Others: 0		CSF leakage: 0 Seizure: 1 Others: 0		CSF leakage: 1 Seizure: 1 2 Others: 0		0.750
Visual outcome	Improved: 13 Stable: 2 Worsened: 0		Improved: 4 Stable: 7 Worsened: 0		Improved: 17 Stable: 9 Worsened: 0		0.006

## Data Availability

Data are available upon request from luigi.sampirisi@gmail.com (accessed on 1 January 2022).

## Supplementary Materials

The following supporting information can be downloaded at: https://www.mdpi.com/article/10.3390/tomography8050197/s1, Video of an extradural clinoidectomy performed on a patient included in the present study.
